# An emerging role for microRNA in the regulation of endothelin-1

**DOI:** 10.3389/fphys.2013.00022

**Published:** 2013-02-19

**Authors:** Mollie E. Jacobs, Charles S. Wingo, Brian D. Cain

**Affiliations:** ^1^Cain Laboratory, Department of Biochemistry and Molecular Biology, University of FloridaGainesville, FL, USA; ^2^Department of Medicine, University of FloridaGainesville, FL, USA; ^3^Malcom Randall North Florida/South Georgia VA Medical CenterGainesville, FL, USA

**Keywords:** microRNA, endothelin-1, endothelin signaling, EDN1 mRNA, ET-1

## Abstract

Endothelin-1 (ET-1) is a peptide signaling molecule serving diverse functions in many different tissues such as the vasculature and the kidney. The primary mechanism thought to control ET-1 bioavailability is the rate of transcription from the ET-1 gene (*EDN1*), but recent research suggests that *EDN1* expression is attenuated by microRNA (miRNA)—mediated regulation. The action of specific miRNAs on *EDN1* mRNA appears to vary greatly in a tissue specific manner. This review provides a summary of our current understanding of miRNA-*EDN1* interaction.

## Introduction

Endothelin-1 (ET-1) is an intercellular signaling molecule expressed in many different organ systems and tissues. Although ET-1 is best known as a potent vasoconstrictor, ET-1 plays important roles in the vasculature, kidney, humoral systems, nervous system, and in the heart (for review see Kohan et al., 2011). For example, in the renal collecting duct ET-1 is an effector of open channel probability for the epithelial sodium channel (Bugaj et al., 2008). Blocking ET-1 action by collecting duct specific knockout of either ET-1 or the endothelin B receptor drives elevated blood pressure with increased sodium load (Bugaj et al., 2008, 2012). The ET-1 gene (*EDN1*)^1^ is equipped with an array of transcriptional regulatory elements that are activated in response to differing stimuli in a wide variety of cell types (Stow et al., 2011; Welch et al., 2013). Again in the renal collecting duct, the *Edn1* gene is regulated by aldosterone through the mineralocorticoid and glucocorticoid receptors (Stow et al., 2009, 2012), and independently by calcium via the nuclear factor of activated T-cells (NFAT) (Strait et al., 2010). Regulation of *EDN1* occurs primarily at the level of transcription, however, it is becoming clear that *EDN1* mRNA is regulated at the post-transcriptional level. This regulation is reflected in the relative instability of the *EDN1* mRNA, with a measured half life of approximately 15 min (Inoue et al., 1989).

The mechanisms providing this apparent lability appear to be focused on the 3′ untranslated region (UTR) of the *EDN1* mRNA. In humans and other mammals, the 3′UTR represents over 50% of the total mRNA length and contains long tracts of highly conserved sequence. Alignment of 19 species of class Mammalia yielded greater than 80% sequence identity between *EDN1* 3′UTRs, and this conservation extends more broadly among vertebrate species. The level of conservation by itself suggests that there are elements in the 3′UTR that are critical for tight regulation of *EDN1* mRNA availability. The primary focus of this review is to consider microRNA (miRNA) action related to expression and function of ET-1.

Although the emphasis is miRNA-mediated regulation, there are certainly other post-transcriptional mechanisms acting on *EDN1* mRNA. Mammalian *EDN1* 3′UTRs typically contain 3–7 AU-rich elements (AREs) depending on the species. Early work by Mawji et al. (2004) identified one human ARE (position 978–987) that facilitated mRNA turnover via the AUF1-proteosome pathway. However, the AREs were not sufficient to fully destabilize the *EDN1* message. The importance of apparent ARE action was supported by additional studies implicating the same region in *EDN1* lability (952–991) (Reimunde et al., 2005). While these studies demonstrated that AREs play a role in *EDN1* mRNA turnover, they also suggested that the instability of the *EDN1* mRNA was not entirely dependent on the AREs.

## miRNA regulation of EDN1 expression

**Figure 1:**
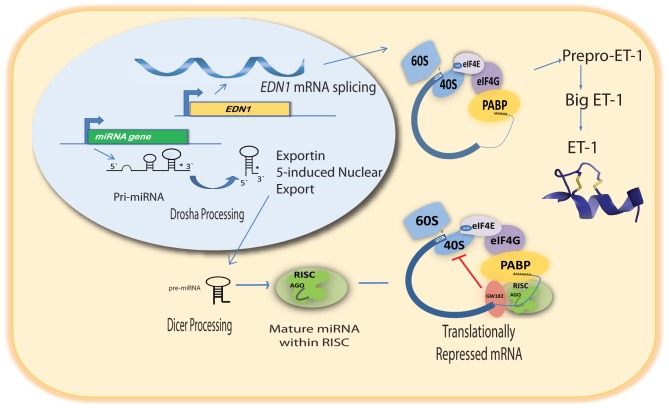
**Pathway of miRNA-mediated action on *EDN1* mRNA**.

**Figure 2:**
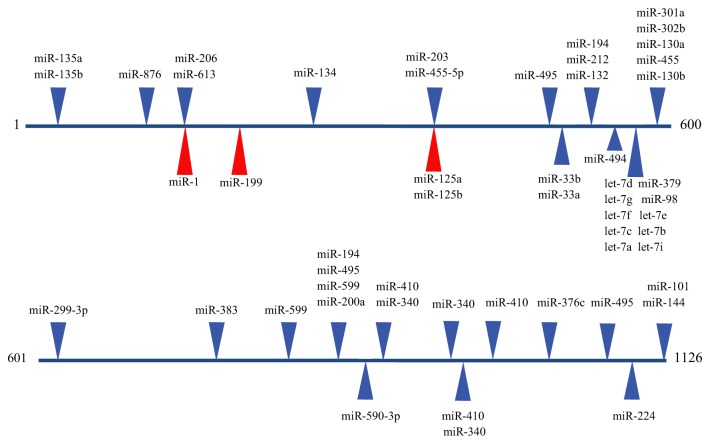
**Predicted miRNA binding sites in the human *EDN1* 3′UTR.** Predicted miRNA target sites within the human *EDN1* 3′UTR (NM_001955) were determined using microRNA.org (Betel et al., 2008). Depicted are target sites of conserved miRNAs with high probability mirSVR scores (blue arrows), and target sites for miRNAs empirically shown to interact with *EDN1* mRNA (red arrows).

The emergence of miRNAs as a major gene regulatory mechanism provided a likely candidate for *EDN1* mRNA control. miRNAs are a family of small (18–24 nt), single stranded, endogenously produced, non-coding RNAs. The action of a miRNA is dependent on its incorporation into the RNA induced silencing complex (RISC). A miRNA-RISC indentifies target mRNAs by imperfect base pairing between the miRNA and the mRNA, most often in the 3′UTR. As a result, miRNAs specifically regulate gene expression by blocking protein translation and/or inducing degradation of targeted miRNAs (Figure 1). Clearly, miRNAs play important roles in the regulation of metabolism in the healthy cell, and dysregulation of miRNA levels is associated with the pathogenesis of many diseases (Feng and Feng, 2011; Abdellatif, 2012; Fish, 2012; Ho and Kreidburg, 2012). Examination of the human *EDN1* 3′UTR revealed many predicted miRNA binding sites within conserved sequence segments (Figure 2), suggesting that multiple miRNAs are likely to be targeting the *EDN1* mRNA. Given that ET-1 is expressed in many different tissues, it seems likely that miRNAs may be involved in regulating basal *EDN1* mRNA expression in a tissue-specific manner. For example in a murine kidney cell line (mIMCD-3), microarray analysis indicated that 12 miRNAs predicted to target *Edn1* mRNA are expressed in high abundance (Jacobs et al., manuscript in preparation).

An excellent example of tissue specific miRNA action on *EDN1* expression can be seen in endothelial biology. Yeligar et al. (2009) studied liver sinusoidal endothelial cells (rLSEC) derived from ethanol-fed rats. These cells displayed large increases of *Edn1* mRNA in comparison to non-ethanol controls. Two miRNAs, miR-155 and miR-199, showed decreased expression in response to ethanol treatment. A binding site for miR-199 (position 166) was found in the human and rat 3′UTR of the *EDN1* mRNA (Figure 2). Overexpression of either miRNA completely inhibited ethanol-induced *EDN1* mRNA expression. Similar results were obtained with human microvascular endothelial cells (HMEC-1). Inhibiting miR-199 levels led to an increase in ET-1 protein levels in the presence of ethanol. The data provided convincing evidence that miR-199 regulates ethanol-induced ET-1 levels in both rLSEC and HMEC-1.

Another study examined two miRNAs, miR-125a and miR-125b, that are endogenously expressed in abundance in vascular epithelial cells (Li et al., 2010). Both miRNAs are predicted to target the *EDN1* 3′UTR at an element located at position 373 (Figure 2). 293A cells containing Luciferase-*EDN1* 3′UTR reporter vectors were co-transfected with either miR-125a or miR-125b overexpression plasmids. As expected for miRNAs targeting *EDN1* expression, both miRNA over-expression plasmids suppressed luciferase expression in a dose-dependent manner. Interestingly, in vascular epithelium the action of these miRNAs are regulated by oxidized low-density lipoprotein (oxLDL), which was shown to increase prepro-ET-1 production. miR-125a expression was enhanced by oxLDL treatment and miR-125b expression simultaneously decreased. The authors suggested that this could be an example of coordinate miRNA-mediated *EDN1* mRNA regulation.

Recently, Li et al. (2012a) proposed a novel role for miRNA regulation of *EDN1*. Levels of miR-1 (Figure 2) increase during cardiac and skeletal muscle development, while ET-1 protein levels significantly decrease during differentiation of DMSO-induced P19 teratoma cells to cardiomyocytes (Monge et al., 1995). Several murine and human tissues and cell lines were examined for levels of both miR-1 and *EDN1* mRNA. In tissues where miR-1 levels were high, such as cardiac and skeletal muscle, *EDN1* mRNA levels were low. In contrast, tissues that had high levels of *EDN1* mRNA expression, including the lung and kidney, had low levels of miR-1 expression. The concept of a negative correlation between miR-1 and *EDN1* mRNA gained additional support from a series of luciferase reporter assays showing overexpression of miR-1 action on the *EDN1* 3′UTR in 293 T cells. miR-1 may also be regulating *EDN1* mRNA levels in some hepatocarcinomas. miR-1 is known to act as a tumor suppressor in several types of cancer (Rao et al., 2010; Hudson et al., 2011). Additionally, silencing the miR-1 gene was shown to induce proliferation of hepatoma cells (Datta et al., 2008). Li et al. (2012b) demonstrated that miR-1 is down regulated in two different hepatocarcinoma cell lines, Hep2G and Hep3B, relative to an immortalized human liver cell line (LO2). Interestingly, miR-1 overexpression inhibited proliferation in HepG2 and Hep3B cells, and addition of exogenous ET-1after transfection increased cell viability. In many malignant cells upregulation of ET-1 has been shown to promote cell proliferation (Bagnato et al., 2011), so it seems reasonable that the decrease in miR-1 levels in hepatocarcinoma cells is a contributing factor in increased cell proliferation.

The studies described above successfully used a candidate miRNA approach by examining miRNA binding sites predicted to bind a target mRNA based on *in silica* analysis. Unfortunately, prediction of a binding site and presence of a candidate miRNA is not sufficient to assume miRNA action on the target mRNA. For example, the let-7 family of miRNAs are highly abundant in a murine inner medullary collecting duct cell line and have a highly conserved putative binding site in the *Edn1* 3′UTR. Knockdown of let-7c and let-7f with anti-miRs had no obvious effect on *Edn1* mRNA levels (unpublished data). An anti-miR is a small synthetic chemically modified single stranded RNA that base pairs to the mature miRNA to block its action. This type of negative results emphasize the need for empirical testing rather than reliance on computer analysis in assessing miRNA action.

In diabetes, glucose has been shown to increase the levels of several vasoactive factors, including ET-1(Feng and Chakrabarti, 2012). It has been suggested that the resulting elevation of these factors contributes to the tissue damage seen in organs affected by diabetic complications. An elegant study focused on the role of miR-320 in diabetes (Feng and Chakrabarti, 2012). To determine if miR-320 was regulated by glucose, streptozotocin-diabetic (STZ) rats were fed either a control or high glucose diet. One month after the onset of diabetes there was a significant decrease in miR-320 expression in STZ diabetic rat cortical tissue. Next, human umbilical vein endothelial cells (HUVEC) were treated with high levels of glucose and as expected *EDN1* mRNA levels increased. HUVECs were then transfected with a miR-320 mimic to specifically block miR-320 binding to target mRNAs. A mimic is a small double stranded chemically modified synthetic RNA designed to bind to a target mRNA resulting in RISC-based decrease in target mRNA expression. The level of *EDN1* mRNA and ET-1 was significantly decreased when HUVECs treated with glucose were also transfected with a miR-320 mimic. This suggests that miR-320 plays a role in post-transcriptional regulation of ET-1 and other vasoactive factors. Thus, in diabetes, the downregulation of miR-320 may lead to the upregulation of those factors contributing to the pathogenic state.

## ET-1 impact on miRNA levels

Up to this point, our focus has been on miRNA action on *EDN1* expression. However, it is also becoming clear that ET-1 itself may be causing changes that influence the miRNA content in cells. For example, ET-1 has been shown to activate monocytes, leading to an increase the expression of the chemokine macrophage inflammatory protein-1β (MIP-1β) (Gonsalves and Kalra, 2010). Patients with sickle cell disease exhibit increased levels of circulating proinflammatory cytochemokines. Work by Gonsalves and Kalra (2010) examined the effect of ET-1 on miRNA expression in a human acute monocytic leukemia cell line (THP-1). In this study, miRNAs with putative binding sites within the MIP-1β 3′UTR that were known to be upregulated in cancer or under hypoxic conditions were selected for investigation. In THP-1 cells treated with ET-1, 60–80% reductions were seen in several miRNAs, including miR-20, miR-194, and miR-195a, relative to untreated cells. miR-195a was chosen for further examination because of a highly conserved binding site in the MIP-1β 3′UTR. Treatment of THP-1 cells with anti-miR-195a prior to ET-1 treatment resulted in a dramatic increase in MIP-1β levels, this increase was reduced to basal levels when a miR-195a overexpression plasmid was transfected prior to ET-1 treatment. In primary human blood monocytes only a modest increase was seen when anti-miR-195a was transfected prior to ET-1 treatment, and this increase was attenuated by a miR-195a overexpression plasmid. This change in mRNA corresponded with a change in ET-1 induced-MIP-1β protein levels inTHP-1 cells. These findings suggested that miR-195a is a negative regulator of ET-1-induced MIP-1β mRNA and protein expression.

Another example of how ET-1 stimulus can affect miRNA levels can be seen in a cardiac-specific miR-23a transgenic mouse (Wang et al., 2011). These mice developed normally to adulthood and did not exhibit any substantial defects in cardiac function or morphology. However, an exaggerated hypertrophic response developed when animals were treated with phenylephrine. They also displayed an increase in heart/body weight ratio, increased cardiomyocytes size, and elevated levels of hypertrophic specific markers. The levels of miR-23a were shown to significantly increase in response to ET-1 treatment, and knockdown of miR-23a attenuated the hypertrophic responses induced by ET-1. Additionally, miR-23a targets the transcription factor Foxo3a. Foxo3a inhibits cardiac hypertrophy, and ET-1 was shown to induce a decrease in Foxo3a levels. Looking at a downstream target of Foxo3a, magnesium superoxide dismutase (MnSOD), ET-1-induced reduction of MnSOD was attenuated by transfection of anti-miR-23a. These results led to the suggestion that ET-1 initiates hypertrophy through a miR-23a-Foxo3a pathway.

A unique relationship between ET-1 signaling and primary miRNA (pri-miRNA) regulation has been observed by von Brandenstein et al. (2011). Under control conditions, high nuclear levels of protein kinase Cα (PKCα) bind to pri-miR-15a and prevent the release of miR-15a in the Caki-1 renal cell carcinoma cell line. However, ET-1 stimulation caused a drop in nuclear PKCα levels and preliminary-miR-15a was exported from the nucleus and processed into the mature form of miR-15a. Blocking either endothelin receptor decreased nuclear levels of PKCα and significantly decreased levels of mature miR-15a. miR-15a regulation by PKCα is seen in several other ET-1 inducible cell lines derived from malignant tumors, namely a melanoma cell line (SKmel 28) and a breast carcinoma cell line (MCF-7). Therefore, depression of miR-15a may represent an important mechanism of action for ET-1 signaling in tumor biology.

## Future directions

The studies described here have laid the ground work demonstrating that miRNAs act on *EDN1* mRNA in many different cells, such as principal cells of the renal collecting duct. However, observations of miRNA action on ET-1 are isolated, reflecting the function of individual miRNAs in a few selected cell types and tissues. An area open to investigation is how changes in the cellular environment affect the miRNA content. What is the impact of hormonal or mitogenic stimuli on the miRNA content in a cell? In turn, how do the changes in the miRNA landscape affect *EDN1* mRNA levels? We already know that stimulation of cells with ET-1 can cause changes in the expression levels of several different miRNAs. Since ET-1 can function by an autocrine mechanism, ET-1 has the potential to indirectly affect its own expression via miRNA. Finally, caution needs to be taken in attributing miRNA affects to a direct action, because one miRNA can target many different mRNAs. This raises the specter of off target effects in the use of miRNA overexpression and inhibition. It seems reasonable to expect that the next generation of papers will adopt more comprehensive technologies to investigate miRNA action on expression of ET-1 and miRNA-mediated effects of ET-1. Hopefully defining the relationship between ET-1 and miRNAs will contribute to an understanding of the pathophysiology of ET-1 dysregulation.

It has been well established that miRNAs contribute to the development of a healthy kidney. Podocyte specific knockout of Dicer results in proteinuria, and mutants progressed rapidly to end stage kidney disease (Harvey et al., 2008). Deletion of Dicer from the ureteric bud and its descendents resulted in severe unilateral or bilateral hydronephrosis by 3 months (Pastorelli et al., 2009). While it is clear that miRNAs are required for normal function of the kidney, it is equally apparent that alteration of miRNAs occurs in many renal diseases. In polycystic kidney disease the downregulation of miR-15a is thought to contribute to *in vitro* cystogenesis by targeting the cell cycle regulator Cdc25A (Lee et al., 2008). Interestingly, profiling renal biopsies from patients with either immunoglobulin A nephropathy (Dai et al., 2008a) or lupus nephritis (Dai et al., 2008b) using miRNA microarrays showed that in both cases over 60 miRNAs were differentially expressed, and in each case roughly half of those miRNAs were downregulated. Work by Juan et al. (2010) identified a panel of 35 miRNAs that can distinguish clear-cell type renal cell carcinoma samples from patient matched normal kidney tissue with high confidence. These studies highlight the need to define the miRNA landscape, with the potential for developing molecular markers for early detection and diagnosis.

Understanding the interplay between miRNAs and *EDN1* mRNA has the potential for new clinical applications. Currently, there are multiple clinical trials examining miRNA profiles in many disease states with the aim of identifying miRNAs that could be used for prognostic or diagnostic purposes (clinicaltrials.gov). Other studies are examining the change in miRNA content in response to therapy. Furthermore, in a recent study by Lanford et al. (2010), treatment of hepatitis C virus (HCV)-infected primates with anti-miR-122 resulted in a long-lasting suppression of hepatitis C viremia, a downregulation of interferon-related genes, and improvement of HCV-induced liver pathology. Importantly, there was no evidence of viral resistance or any side effects in the treated animals. This represents a critical step toward development of miRNA-targeted therapies. We are at the dawn of an age of using our understanding of miRNA functions and interactions as a guide for treatment. Elucidating the complex relationship between miRNAs and ET-1 will contribute to the development of targeted therapies where ET-1 dysregulation leads to pathological phenotypes.

### Conflict of interest statement

The authors declare that the research was conducted in the absence of any commercial or financial relationships that could be construed as a potential conflict of interest.

## Footnotes

^1^By convention the human endothelin-1 gene is written *EDN1*, while the rat and murine endothelin-1 gene are written *Edn1*. In this review, the human 3′UTR will be used to provide sequence coordinates.
